# A deep learning model based on dynamic contrast-enhanced magnetic resonance imaging enables accurate prediction of benign and malignant breast lessons

**DOI:** 10.3389/fonc.2022.943415

**Published:** 2022-07-22

**Authors:** Yanhong Chen, Lijun Wang, Ran Luo, Shuang Wang, Heng Wang, Fei Gao, Dengbin Wang

**Affiliations:** ^1^ Department of Radiology, Xinhua Hospital Affiliated to Shanghai Jiao Tong University School of Medicine, Shanghai, China; ^2^ Department of Medicine, Beijing Medicinovo Technology Co., Ltd., Beijing, China

**Keywords:** DCE MRI, breast cancer, convolution neural networks, deep learning, machine learning

## Abstract

**Objectives:**

The study aims to investigate the value of a convolutional neural network (CNN) based on dynamic contrast-enhanced magnetic resonance imaging (DCE-MRI) in predicting malignancy of breast lesions.

**Methods:**

We developed a CNN model based on DCE-MRI to characterize breast lesions. Between November 2018 and October 2019, 6,165 slices of 364 lesions (234 malignant, 130 benign) in 364 patients were pooled in the training/validation set. Lesions were semi-automatically segmented by two breast radiologists using ITK-SNAP software. The standard of reference was histologic consequences. Algorithm performance was evaluated in an independent testing set of 1,560 slices of 127 lesions in 127 patients using weighted sums of the area under the curve (AUC) scores.

**Results:**

The area under the receiver operating characteristic (ROC) curve was 0.955 for breast cancer prediction while the accuracy, sensitivity, and specificity were 90.3, 96.2, and 79.0%, respectively, in the slice-based method. In the case-based method, the efficiency of the model changed by adjusting the standard for the number of positive slices. When a lesion with three or more positive slices was determined as malignant, the sensitivity was above 90%, with a specificity of nearly 60% and an accuracy higher than 80%.

**Conclusion:**

The CNN model based on DCE-MRI demonstrated high accuracy for predicting malignancy among the breast lesions. This method should be validated in a larger and independent cohort.

## Introduction

Although breast cancer mortality is decreasing, largely owing to improved treatments, breast cancer incidence has been steadily increasing (1). Female breast cancer has now surpassed lung cancer as the leading cause of global cancer incidence in 2020, with an estimated 2.3 million new cases, representing 11.7% of all cancer cases (2). Along with mammography and ultrasound, dynamic contrast-enhanced magnetic resonance imaging (DCE-MRI) plays an integral role in the detection and characterization of breast cancer (3). Although MRI is the most sensitive imaging modality to detect breast cancer, the specificity of breast MRI is only moderate, with positive predictive values of 35–64% for screening MRI in high-risk women (4). Lesion identification can be limited by background enhancement, which may mask or mimic lesions, and many benign lesions also show strong contrast enhancement, leading to a false-positive diagnosis, unnecessary biopsy, or overtreatment. To increase the number of screening and preoperative MRI performed, it is urgent to find an efficient way, such as developing imaging models, to differentiate between benign and malignant lesions to improve the diagnostic accuracy.

Machine learning (ML) is poised to address some or all of these problems. Deep learning (DL) is a subtype of machine learning that uses layers of artificial neurons, called neural networks, to learn. It has turned out to be robust at discovering intricate structures in high-dimensional data and has beaten other machine-learning techniques in many aspects (5). A convolutional neural network (CNN) is a common deep-learning method applied to analyze photographic, pathological, and radiographic images, which is capable of automatically learning features in contrast to those traditional methods where hand-crafted features are used. CNN has frequently been implemented for medical image analysis in various clinical tasks, including segmentation, abnormality detection, disease classification, computer-aided diagnosis, and retrieval (6). For classification tasks, the CNNs take the raw image data as input and extract the features using a hierarchy of layers to learn discriminative patterns. Supervised by the pathological results of the case, the models must predict the likelihood of cancer and non-cancer.

Currently, deep learning has been widely applied to detect and diagnose breast cancer by mammography and has shown promising results (7–11). Studies are limited regarding the use of CNNs for diagnostic classification of lesions in breast MRI (for differential diagnosis of benign vs malignant lesions) due to multiple sets of images with varying tissue contrast and images of DCE-MRI at different times with varying signal intensities (12, 13). In this study, we developed a CNN model for predicting malignancy among the breast lesions based on DCE-MRI.

## Materials and methods

### Study population

This retrospective study was approved by the Institutional Ethics Committee of our hospital and the informed consent requirement was waived (No. XHEC-D-2021-185). Between 1 November 2018 and 31 October 2019, a total of 1,020 lesions of 807 patients were enrolled in the training/validation set. Inclusion criteria were as follows: (1) pathological results were obtained through vacuum-assisted breast biopsy or open surgery; (2) patients underwent breast MRI examination before the operation. Exclusion criteria were as follows: (a) did not exhibit an enhancing lesion on DCE-MRI or with insufficient image quality and/or (b) the patients received any therapy before breast MRI examination. The image quality was evaluated by two experienced radiologists in breast imaging in consensus.

For independent testing, the cases performed from January to December 2014 were collected based on the same selection criteria.

### Imaging acquisition

Imaging was performed on a 3.0 T whole-body MRI scanner (Ingenia, Philips, Netherlands for patients in the training/validation set; Signa HDxt, GE Healthcare, America for patients in the testing set). The patients were positioned in the prone position with both breasts placed in an eight-channel phase-array breast coil. The acquisition parameters of the breast MRI protocol are given in **Table 1**. Details of the MRI protocol have been added in **Appendix 1**.

**Table 1:** Acquisition Parameters Used in the Breast DCE-MRI Protocol.

Parameters	e_THRIVE	VIBRANT
slice thickness/gap, mm	2/0	1.2/0
matrix	407 × 404	416 × 320
FOV, cm	34	38
TR/TE/TI, ms	4.2/2.1/-	4.3/2.1/14
FA	12	10
resolution,mm	0.835 × 0.841	0.913 × 1.188

FOV, field of view; TR/TE, repetition time/echo time; FA, flip angle.

The Volume Image Breast Assessment (VIBRANT) sequence on Signa HDxt and enhanced T1 high-resolution isotropic volume excitation (e_THRIVE) on Ingenia were obtained before and four times after the intravenous injection of Gadopentetate Dimeglumine (Gd-DTPA; Beilu, Beijing, China) with 0.1 mmol/kg at a flow rate of 2 ml/s and a 20 ml normal saline flush.

### Data preprocessing and annotation

**Figure 1:**
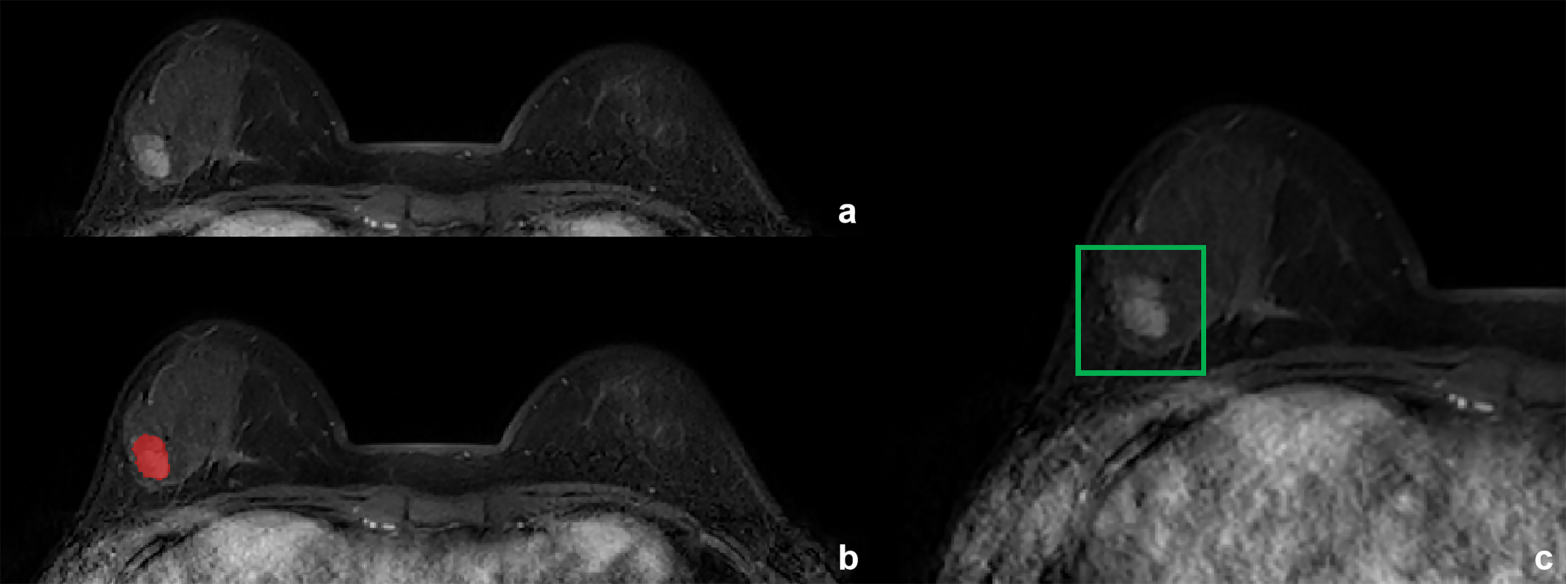
Fibroadenoma in a 29-year-old woman. **(A)** T1-weighted postcontract image. **(B)** Annotation masks (in red) are superimposed over the MR images. The annotations were made by radiologists using ITK-snap. **(C)** The anchor box was generated by the Faster R-CNN model after the segmentation.

**Figure 2:**
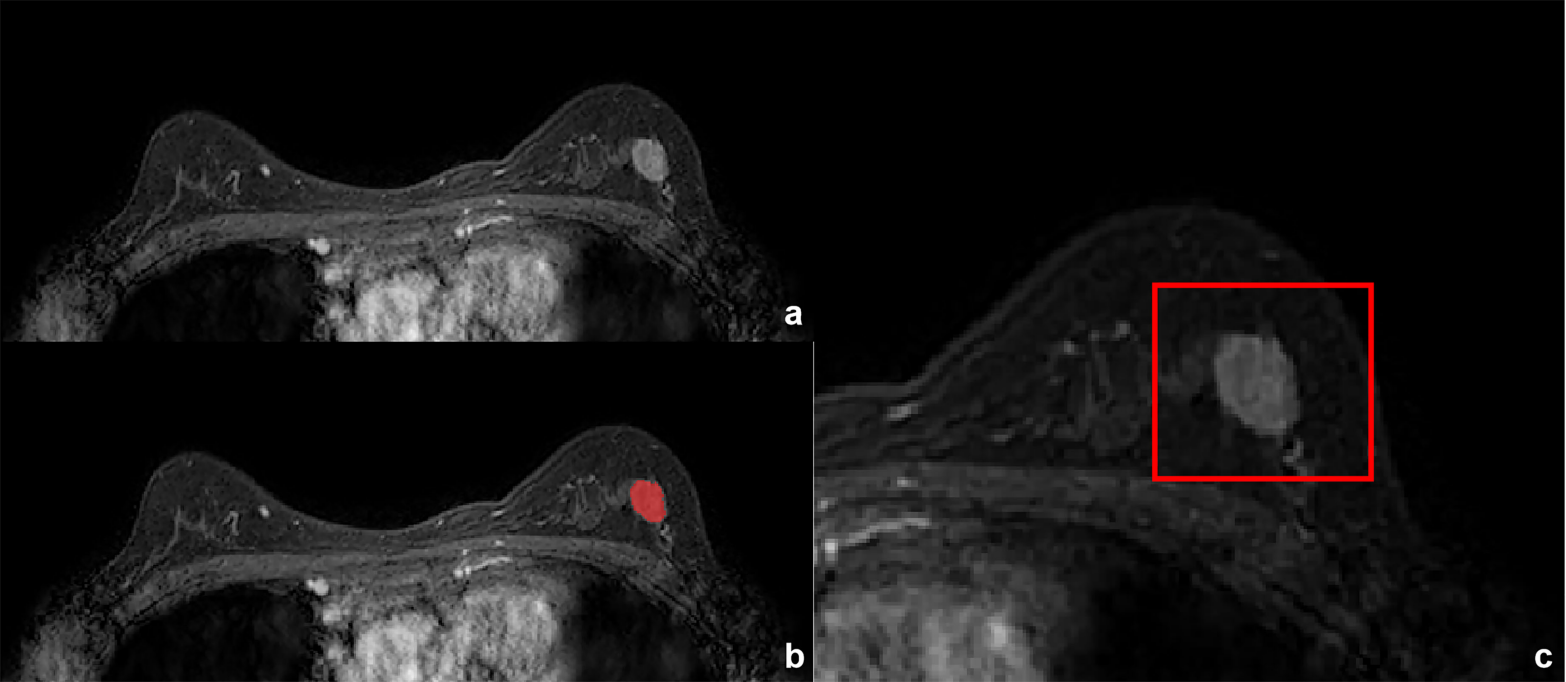
Invasive breast cancer in a 57-year-old woman. **(A)** T1-weighted postcontract image. **(B)** Annotation masks (in red) are superimposed over the MR images. The annotations were made by radiologists using ITK-snap. **(C)** The anchor box was generated by the Faster R-CNN model after the segmentation.

After the digital image and communication in medicine (DICOM) images were obtained, two trained breast imaging radiologists with 9 years and 2 years of work experience subsequently reviewed the MRIs. The lesion area was segmented semi-automatically on the second T1-weighted postcontrast image at a pixel-wise level using ITK-SNAP software (version 3.8, http://www.itksnap.org), an open-source software platform for medical image informatics and analysis. Then, the region of interest (ROI) was manually modified and cross-checked by the two experienced radiologists. Then, the MRIs were reviewed and converted to 16-bit tiff format images. Located at each breast with lesions on the image, a circumscribed square window was cropped and rescaled to 600 × 600 pixel^2^. These square 16-bit tiff images located at the lesion were the input data of the model (**Figures 1**, **2**).

**Figure 3:**
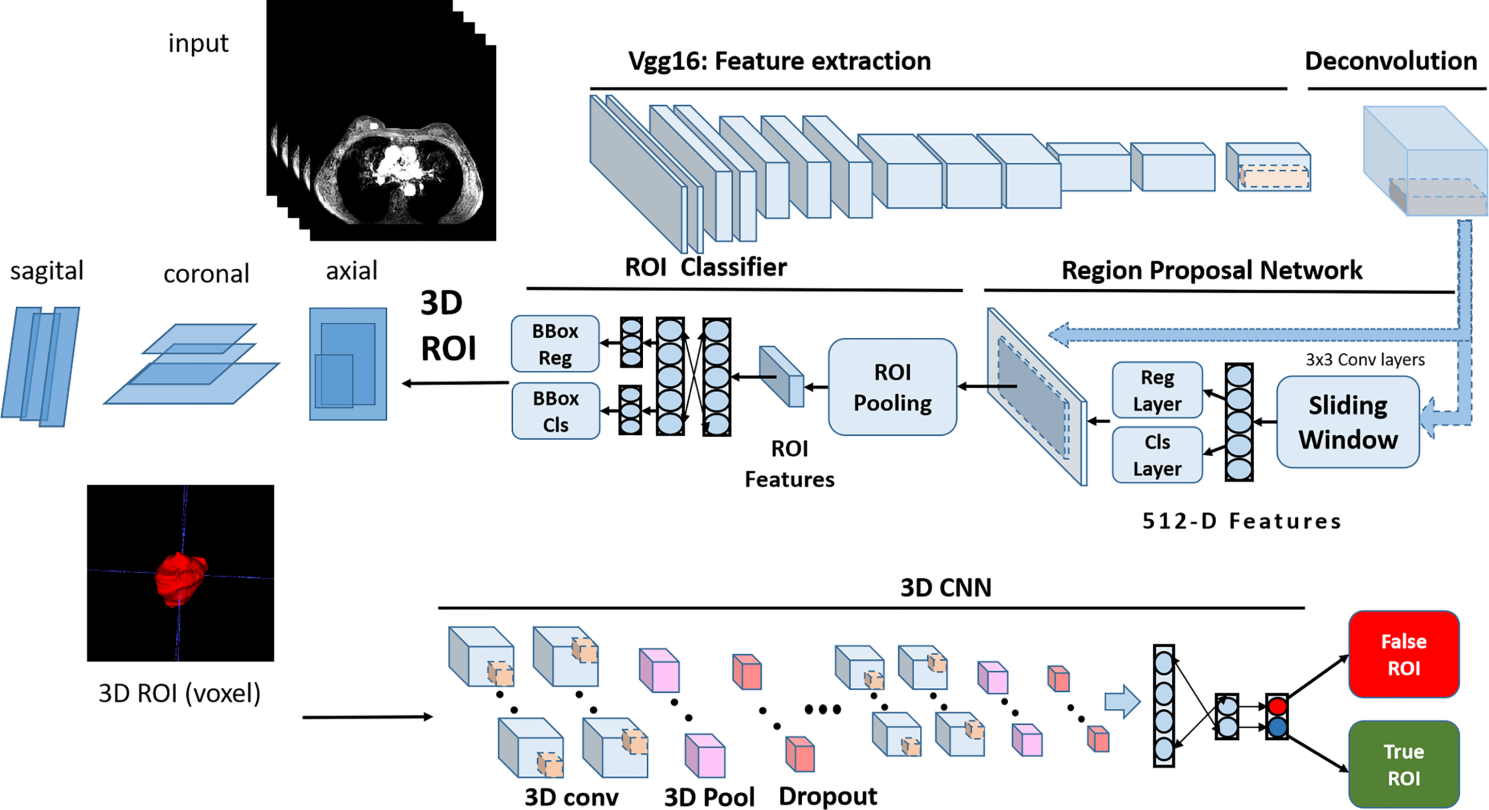
The network architecture of Faster R-CNN for ROI localization.

We stacked the 2D lesion area images of all the slices and obtained a 3D lesion segmentation of the volumetric data. The standards for labeling benign and malignant adopt pathological diagnosis as the gold standard. An automated program was used to generate the anchor box needed by the faster-region based convolutional neural network (Faster R-CNN) model. The anchor box generation algorithm was fixed at square to avoid image feature distortion caused by aspect ratio changes. This was different from the strategy used for processing natural camera images. The algorithm was designed to combine the non-combined areas that belong to the same lesion into one larger anchor box. The network architecture of the Faster R-CNN for ROI localization is shown in **Figure 3**.

### System construction

#### Model structure

**Figure 4:**
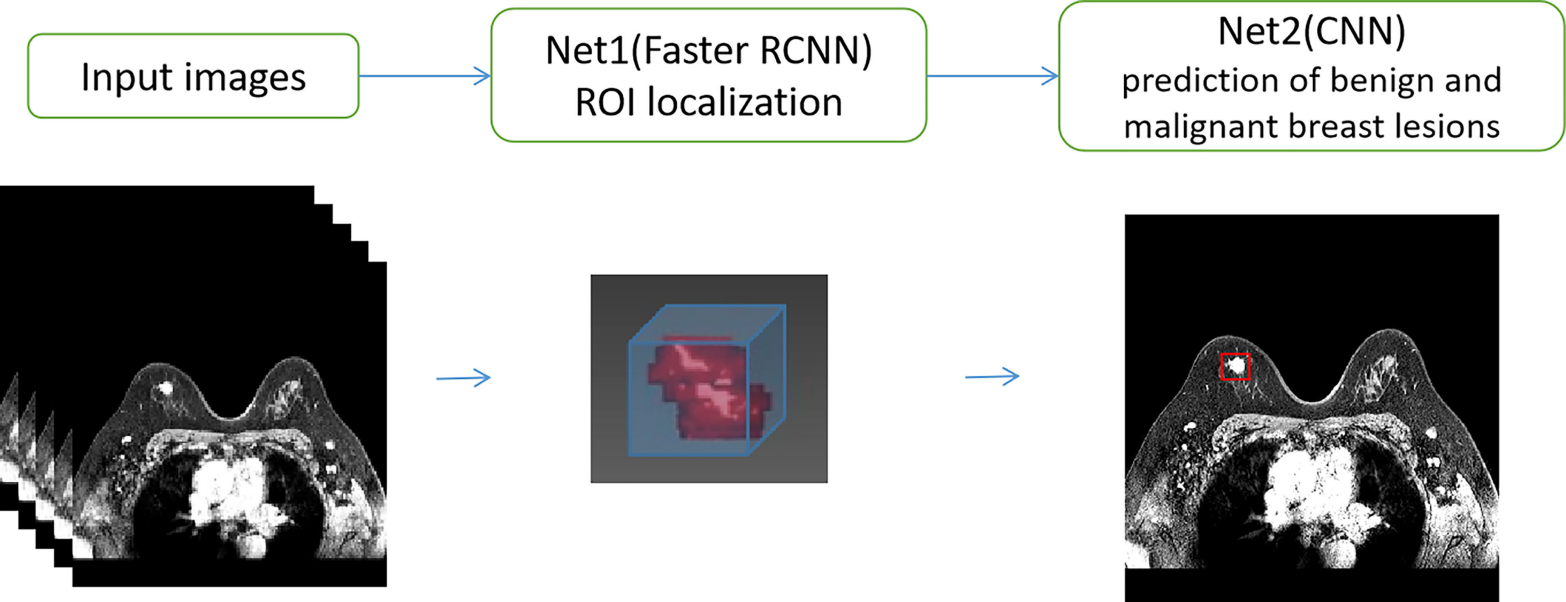
The pipeline of the system consisting of CNN and Faster R-CNN.

**Figure 5:**
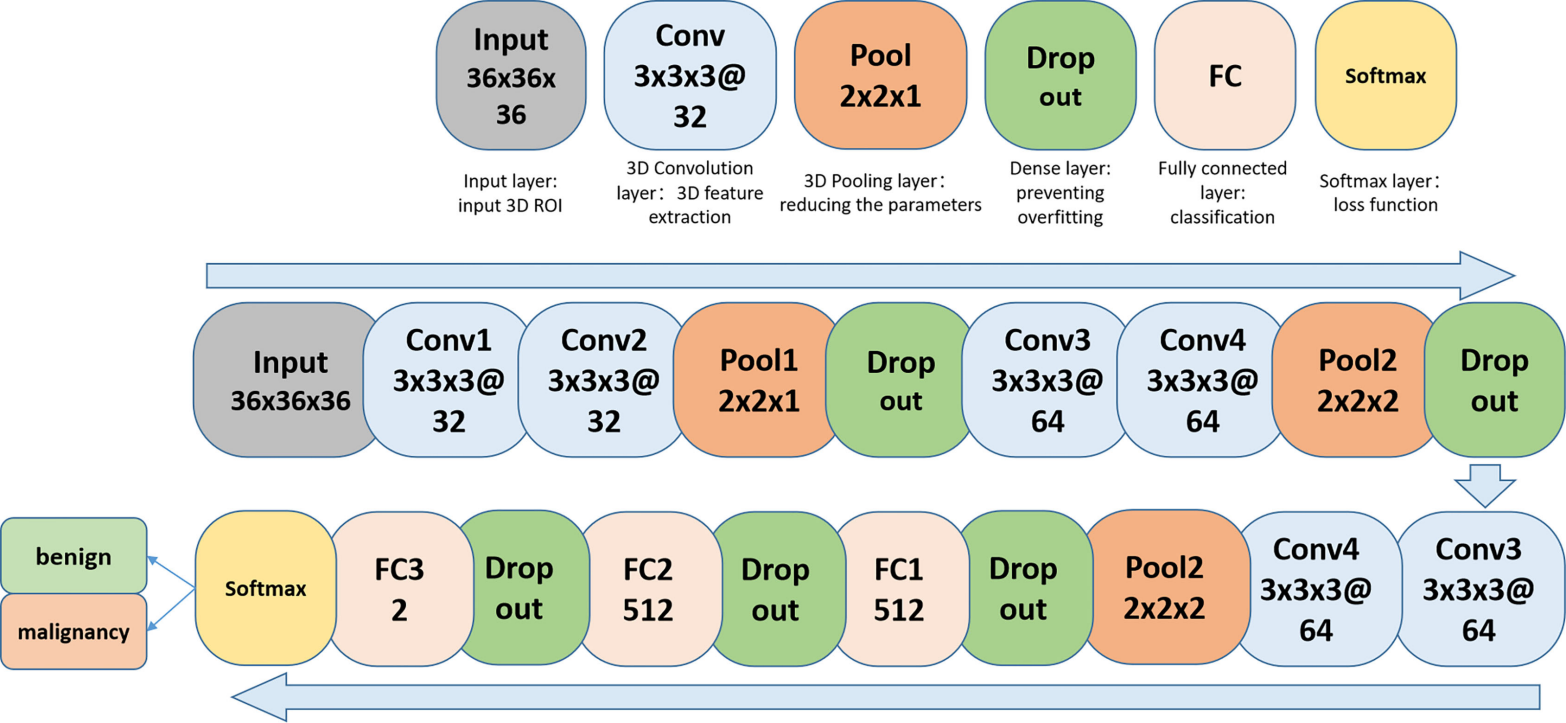
The network architecture of CNN for prediction of benign and malignant breast lesions.

The faster R-CNN, whose backbone CNN adopts the Xception structure, is selected as our developing model. The backbone CNN is followed by a deconvolution layer to expand the feature map and ensure enough detailed features to distinguish between benign and malignant. The input image size of the model is set to 600 × 600. The faster R-CNN model has two outputs. The first output is responsible for locating the ROI. The second output further classified the lesion area as benign or malignant. The pipeline of the system consisting of the CNN and the Faster R-CNN is shown in **Figure 4**. The network architecture of CNN for differentiation between benign and malignant tumors is shown in **Figure 5**.

#### Model training

In the training phase, considering that the amount of training data is insufficient, 5-fold cross-validation is implemented by the scikit-learn package 0.23.2 version. The best model selection is based on the minimum area under the receiver operating characteristic (AUC) metrics as the criterion. Given a three-dimensional affine matrix, random rotating at any angle, scaling with a magnification of 0.8–1.2, and shifting within 10 pixels were used for data augmentation. Approximately 70% of the training MRIs were extracted to apply data augmentation to avoid bias of the model toward the recognition of augmented data over real data.

#### Software and hardware used for development

We constructed, trained, and evaluated the model using Python with TensorFlow-Gpu 2.0 version. The development computer had two NVIDIA 1080Ti graphic cards and an i7-8800k Intel CPU.

### Predictive performance and statistical analysis

The acquisition of the testing dataset was different from the training/validation dataset. In order to prevent the excessive generalization errors caused by different data sources, we fine-tuned the model with a part of the testing dataset, which is a common method used in the computer vision field. Our fine-tuning method was as follows: we combined the fine-tuning dataset with the original training/validation dataset to form a new training/validation dataset, loaded the trained model parameters as initial values, reduced the learning rate by an appropriate multiple (in our cases, 3 is the best from grid search), and retrained all the parameters with the previous model parameters as the initial values.

Then we tested the fine-tuned model on the testing dataset and used two statistical methods: slice-based and case-based. The slice-based method is the direct model execution, and the case-based method is closer to the actual diagnosis process.

The main performance of the models to differentiate between benign and malignant was assessed by AUC. Furthermore, the accuracy, sensitivity, and specificity for diagnosing breast cancer cases and images were evaluated. The Chi-square test or Fisher’s exact test were used to evaluate the differences for categorical variables. Intergroup comparison was performed using the Mann–Whitney U-test for continuous data with a non-normal distribution. All the statistical analyses were performed *via* SPSS 25.0 (IBM) and MatLab. A two-sided P-value of <0.05 was considered statistically significant.

## Results

### Patient characteristics

Finally, a total of 364 lesions in 364 patients (mean age: 52.3 ± 15.5 years, age range: 12–88 years) were used in the training/validation dataset, including 234 malignant lesions and 130 benign lesions. One hundred and twenty-seven lesions in 127 patients (mean age: 51.7 ± 14.6 years, age range: 17–79 years) were used in the testing set, including 85 malignant lesions and 42 benign lesions. The size of the lesions in the training/validation set and the testing set was 2.40 ± 1.39 cm and 2.10 ± 0.98 cm, respectively (*P* = 0.106). The major histological types and enhancement types on MRI of all lesions are listed in **Table 2**.

**Table 2:** Pathology Type and Enhancement Type of Lesions Included in the DataSet.

Lesion type	Training/Validation dataset	Testing dataset	*P*-value
Malignant lesions	234	85	0.591
Invasive ductal cancer	187	71	
Ductal carcinoma *in situ*	15	3	
Mucinous carcinoma	9	2	
Other invasive cancer	23	9	
Benign lesions	130	42	
Adenosis	11	7	
Fibroadenoma	91	34	
Intraductal papilloma	15	1	
other benign lesions	13	0	
Enhancement type			0.688
Mass	304	108	
Non-mass	60	19	
Total	364	127	

### Performance of the CNN models

A total of 6,165 slices of 364 lesions were collected for the training/validation dataset, which was used to adjust the parameters to train a robust model. The testing dataset consisted of 1,403 slices from 127 lesions in order to assess the performance and test the generalization ability of the model.

#### Slice-based

**Figure 6:**
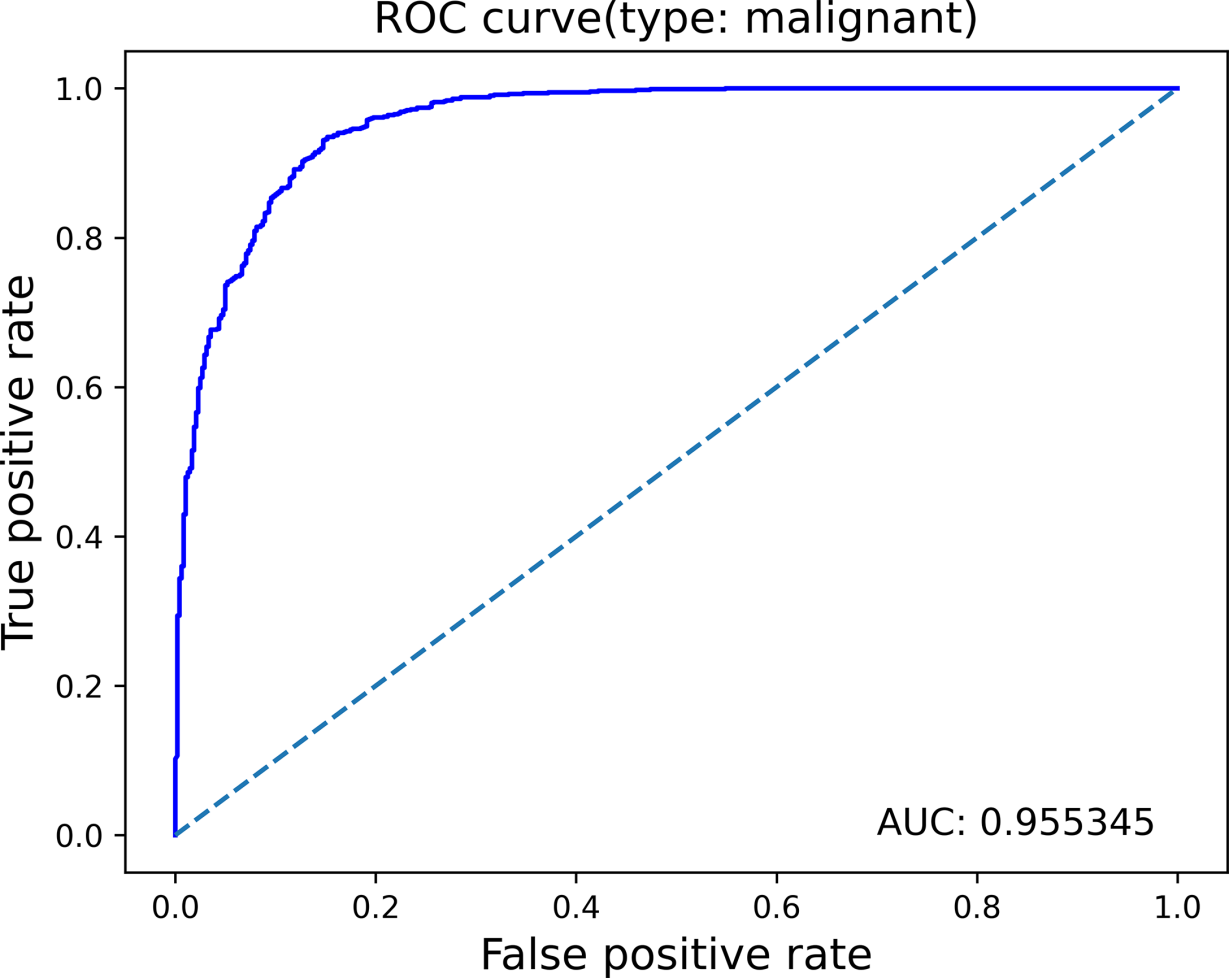
ROC curves for benign vs. malignant classification.

In the testing set, the algorithm performance for breast cancer diagnosis showed 90.3% accuracy, 96.2% sensitivity, and 79.0% specificity, with the area under the curve ROC of 0.955 (**Figure 6**).

#### Lesion-based

The lesion-based results of the testing set are shown in **Table 3**. By adjusting the standard of the number of positive slices, the performance of the model changed. When a lesion with three or more positive slices was determined as malignant, the sensitivity was above 90% with nearly 60% specificity and greater than 80% accuracy.

**Table 3:** Diagnostic Performances of the CNN model evaluated in slice-based and case-based method.

Parameter	Slice-based	case-based 1*	case-based 2*	case-based 3*
Accuracy	90.3%	80.4%	78.0%	81.1%
Specificity	79.0%	40.5%	47.6%	59.5%
Sensitivity	96.2%	100.0%	92.9%	91.8%
Positive predictive value	89.8%	77.3%	78.2%	82.1%
Negative predictive value	91.6%	100.0%	76.9%	78.1%

*case-based 1: As long as one slice is positive, the lesion is classified as malignant;

case-based 2: At least two or more slices are positive, the lesion is classified as malignant; case-based 3: At least three or more slices are positive, the lesion is classified as malignant.

#### Diagnostic performances for mass and non-mass enhancement

**Figure 7:**
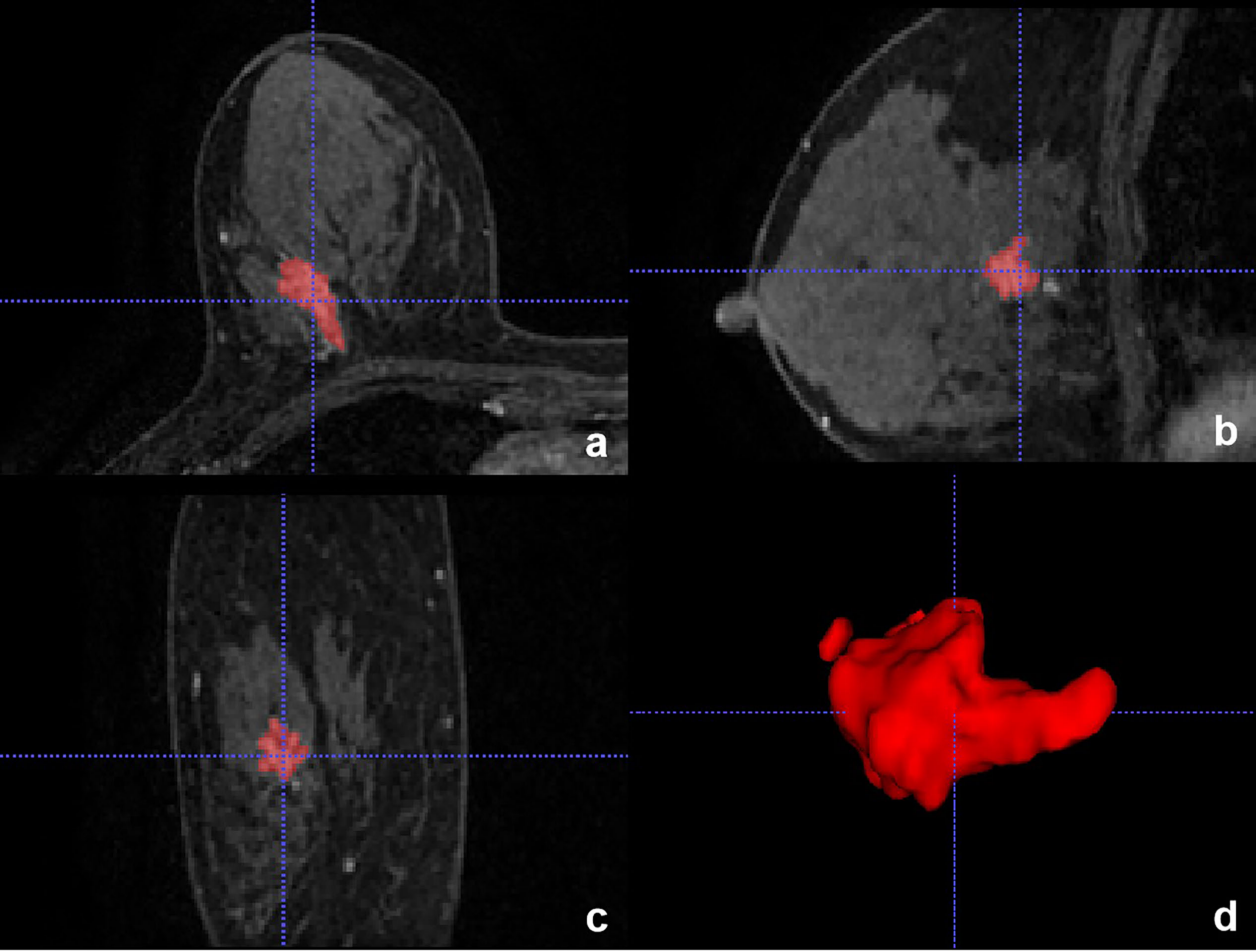
Invasive breast cancer in a 57-year-old woman in the testing set with true-positive result. **(A-C)** Axial, Sagittal, and Coronal T1-weighted postcontract image showed non-mass enhancement in the right breast. **(D)** 3D reconstruction of the lesion.

**Figure 8:**
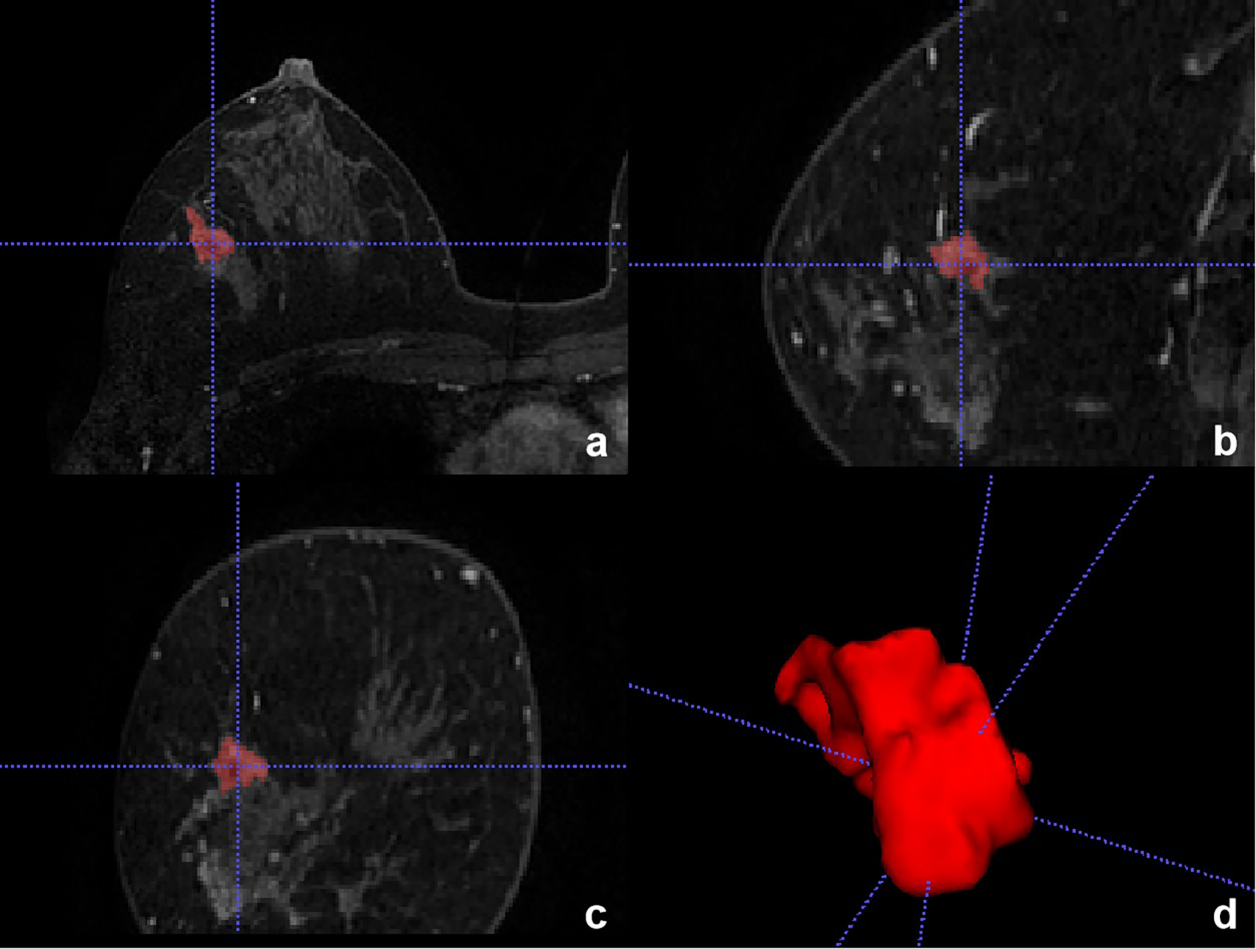
Adenosis in a 46-year-old woman in the testing set with false-positive result. **(A–C)** Axial, Sagittal, and Coronal T1-weighted postcontract image showed non-mass enhancement in the right breast. **(D)** 3D reconstruction of the lesion.

The diagnostic performance of the CNN model for mass and non-mass enhancement is shown in **Table 4**. The negative predictive value (NPV) and specificity for non-mass enhancement were lower than those for mass, while the positive predictive value (PPV), sensitivity, and accuracy for non-mass enhancement were slightly higher than those for mass (**Figures 7**, **8**).

**Table 4:** Diagnostic Performances of the CNN model for mass and non-mass enhancement.

	Mass	Non-mass enhancement
Accuracy	90.2%	91.1%
Specificity	80.9%	0%
Sensitivity	95.9%	97.5%
Positive predictive value	89.1%	93.3%
Negative predictive value	92.5%	0%

## Discussion

Although MRI is a powerful tool for the diagnosis and screening of breast cancer, the utility of breast MRI has been restricted because of the limited availability of sites that could offer this method and the lack of experienced radiologists for interpreting breast MR images. In addition, a suspicious lesion detected by MRI usually needs to be investigated through a biopsy. However, the biopsy is an invasive procedure at risk of infection and misdetection. Continued concerns have been raised regarding potential harm associated with unnecessary biopsies and surgeries that are triggered by imaging findings in patients who do not have breast cancer (14, 15). Our study was to distinguish breast lesions between benign and malignant and prevent unnecessary biopsy by objectively analyzing noninvasive breast MRI images.

Machine learning (ML) is likely to address some or all of these limitations. A machine learning model incorporating the full spectrum of patient data offers a means to distinguish patients with breast cancer from those without and thereby reduce unnecessary surgical interventions. In other words, it can help to improve diagnostic specificity and decrease false-positive interpretations. McKinney et al. (10) reported that the artificial intelligence (AI) system outperformed radiologists during breast cancer screening with a greater AUC by an absolute margin of 11.5% and could reduce the workload of the second reader by 88%. Yala et al. (9) reported that the Deep Learning (DL) model had the potential to improve specificity to 94.2% for triaging the mammogram as cancer-free and reduce the workload by 19.3%. Compared with other machine learning methods, one advantage of CNN models is their capability in image analysis (deep feature extraction) and prediction algorithm construction, thereby precluding the need for separate steps of extracting hand-crafted radiomic features and using them as an input for an algorithm to construct a prediction model. Another advantage is the ability to learn complex datasets and achieve high performance without prior feature extraction. For dealing with lesion localization, a faster-region based convolutional neural network (Faster R-CNN), which has the advantage of automatic image segmentation—defining the tumor bounding box (16–18), has been used for identifying the contour of the tumor area before lesion classification. Due to the complex construction, a novel diagnostic system that integrates CNN and the Faster R-CNN model is proposed in our study.

In this study, our CNN faster-RCNN-based diagnostic system demonstrated the potential to distinguish between malignant and benign breast lesions and was successfully validated using MRI conducted by another 3.0T system (Signa HDxt; GE Healthcare). Several novel aspects of our study should be emphasized. First, this is the first report that describes the development of CNN and Faster R-CNN models to facilitate the diagnosis of breast tumors. Second, we stacked the expert-labelled 2D slices of one lesion and obtained a 3D lesion structure to extract volumetric features, which are as important as local texture features in recent literature reports for improving diagnostic performance (19). The generated heat maps made it possible to better understand how the model extracted characteristics, including misclassification. Additionally, with a stable algorithm framework, there is no subjective bias in the image recognition process. The recognition result is stable and does not change with time and operating factors, which can provide a reliable reference for radiologists to reduce variation.

It is worth highlighting that our model had high sensitivity (96.2%) and a large AUC (0.955) for the classification of breast tumors in the external validation, which demonstrated the potential for adaptability and robustness. The fact is that different pulse scan sequence protocols of institutions vary, and in the previous study, most radiomics or deep learning models are trained and tested within the same source dataset. In this study, we used a separate dataset for independent testing. The images of the training set and testing set were acquired from two different machines (Philips vs. GE), both with a standardized protocol, and all results suggest that deep learning can achieve high accuracy and has the potential for clinical implementation.

Several limitations of our study should be considered. First, the specificity of the model was relatively low when distinguishing breast lesions rather than images, due to the high proportion of images of malignant lesions in the dataset. Additionally, the NPV and specificity of the model for non-mass enhancement were low, which was because there were only three benign non-mass enhancements in the testing set, and the model predicted that they were all malignant. Second, we only used DEC-MR images in this study. Recently, multiparametric MRI has also been studied with DL (13, 20). A combined DL model developed incorporating mammography and clinical variables also showed promising results (11, 21). More risk factors such as clinical information, including age, family history of breast cancer, menopausal status, and multiparametric MRI features could be used in future studies. Third, because of the architecture of the CNN, the internal parameters or weights of each layer were invisible. Therefore, the fact that similar frames, even neighboring frames, are classified into different categories cannot be explained exactly. Although the precise reason for this remains unclear, this type of incorrect classification might be due to the limited ability of the CNN used in the current study, which will be more sophisticated in the future.

In conclusion, our trained system rendered a promising performance in classifying the breast lesions into benign or malignant, highlighting its potential for future application as a new tool for clinical diagnosis. The automatic methods can help improve diagnostic accuracy by decreasing interobserver variations, reducing the number of false-positive biopsies and the burden of radiologists. The workload reduction could free radiologists to provide more patient care and perform guided procedures. The number of cases in the database is expected to increase, and the hyperparameters in deep learning are expected to be more optimized, which will further increase the accuracy of the model.

## Data availability statement

The data analyzed in this study is subject to the following licenses/restrictions: The datasets presented in this article are not readily available “Due to the privacy of patients, the MRI data and clinical information related to patients cannot be available for public access”. Requests to access the datasets should be directed to the corresponding author. Requests to access these datasets should be directed to wangdengbin@xinhuamed.com.cn.

## Ethics statement

The studies involving human participants were reviewed and approved by the Ethics Committee of Xinhua Hospital Affiliated to Shanghai Jiaotong University School of Medicine. The patients/participants provided their written informed consent to participate in this study. Written informed consent was obtained from the individual(s) for the publication of any potentially identifiable images or data included in this article.

## Author contributions

YC: study concept, acquisition, analysis and interpretation of data, statistical analysis, and drafting of the article. LW and RL: acquisition, analysis and interpretation of data, statistical analysis, and drafting of the article. HW, SW, and FG: acquisition and analysis of data. DW: study concept and design, analysis and interpretation, and critical revision of article. All authors revised the manuscript critically for important intellectual content and approved the final submitted version.

## Funding

This study was supported by the National Nature Science Foundation of China (Nos. 82071870 and 82101991), the Program of Shanghai Science and Technology Committee (No. 21s21905000), the Special Research Program of Shanghai Municipal Commission of Heath and Family Planning on medical intelligence (No. 2018ZHYL0108), and the Project of Shanghai Municipal Health Commission (No. 20184Y0030). The funders had no role in study design, data collection and analysis, decision to publish, and preparation of the manuscript.

## Conflict of interest

Authors SW, HW, and FG were employed by the company Beijing Medicinovo Technology Co., Ltd.

The remaining authors declare that the research was conducted in the absence of any commercial or financial relationships that could be construed as a potential conflict of interest.

The handling editor XC declared a shared parent affiliation with the author(s) YC, LW, RL, and DW at the time of review.

## Publisher’s note

All claims expressed in this article are solely those of the authors and do not necessarily represent those of their affiliated organizations, or those of the publisher, the editors and the reviewers. Any product that may be evaluated in this article, or claim that may be made by its manufacturer, is not guaranteed or endorsed by the publisher.

## Abbreviations

AUC, Area under the receiver operating characteristic curve; BI-RADS, Breast Imaging Reporting and Data System; CNN, Convolution neural network; DL, Deep learning; ML, Machine learning; MRI, Magnetic resonance imaging; NPV, Negative predictive value; PPV, Positive predictive value.
