# Supplementary material for: A deep learning model based on dynamic contrast-enhanced magnetic resonance imaging enables accurate prediction of benign and malignant breast lessons
Source: Front Oncol. 2022 Jul 22;12:943415. doi: 10.3389/fonc.2022.943415 (PMC9353744; doi:10.3389/fonc.2022.943415)
Supplement: Supplementary file 1 [file DataSheet_1.docx]

**Appendix 1**

For the 3.0T GE MRI scanner (Signa HDxt; GE Healthcare), axial short tau inversion recovery (STIR) was performed firstly (TR/TE/TI, 7060/35.2/170 ms; slice thickness/gap, 4/1 mm; flip angle, 90^。^; FOV, 32cm; and matrix, 320×192). Next, axial diffusion-weighted imaging (DWI) was obtained with b-values of 0 and 800 s/mm^2^ (TR/TE, 5125/66.4 ms; slice thickness/gap, 4/1 mm; flip angle, 90^。^; FOV, 32cm; and matrix, 128 x 128). Then, the dynamic imaging with Vibrant sequence was performed before and five times immediately after the injection of Gadopentetate dimeglumine (Beilu, Beijing, China) with 0.1 mmol/kg as a bolus at a flow rate of 2 mL/s. The parameters of the dynamic sequence were as follows: TR/TE/TI, 4.3/2.1/14 ms; slice thickness/gap, 1.2/0 mm; flip angle, 10^。^; FOV, 38cm; matrix, 416 x 320; and resolution 0.913mm x 1.188 mm. For each phase, the acquisition time was 60s and the center of k-space acquisition time was 30 s. Contrast enhanced images were acquired at 30, 90, 150, 210, and 270 seconds after contrast material injection.

For the 3.0T Philips MRI scanner (Ingenia; Philips Healthcare), axial T2-weighted imaging was performed firstly with the following parameters: TR/TE, 5000/ 65ms; slice thickness/gap, 4/1 mm; flip angle, 90^。^; FOV, 37.2cm; and matrix, 465 x 381. Next, axial DWI was performed with b-values of 0 and 800 s/mm^2^ (TR/TE, 5100/ 72ms; slice thickness/gap, 4/1 mm; flip angle, 90^。^; FOV, 35cm; and matrix,136 x 140). Then, the dynamic imaging with enhanced T1 high resolution isotropic volume excitation (e_THRIVE) sequence was performed before and four times immediately at the injection of Gadopentetate dimeglumine (Beilu, Beijing, China) with 0.1 mmol/kg as a bolus at a flow rate of 2 mL/s. A meantime of 6s (range 4s - 8s) was needed for the injection of contrast agent according to the weight of the patients. The parameters of the dynamic sequence were as follows: TR/TE, 4.2/2.1ms; slice thickness/gap, 1/0 mm; flip angle, 12^。^; FOV 34 cm; matrix 407 x 404; and resolution 0.835mm x 0.841mm. For each phase, the acquisition time was 65s and the center of k-space acquisition time was 52s. Contrast enhanced images were acquired at about 46, 111, 176, and 241 seconds after contrast material injection.
